# Intestinal Surgery Contributes to Acute Cerebellar Ataxia Through Gut Brain Axis

**DOI:** 10.3389/fneur.2019.00995

**Published:** 2019-09-20

**Authors:** Jie Yu, Yuanming Fan, Li Wang, Yanjuan Huang, Jingyi Xia, Le Ding, Chun-Feng Wu, Xiaopeng Lu, Gaoxiang Ma, Samuel Kim, Guo Zheng, Hu Guo, Gang Zhang

**Affiliations:** ^1^Department of Neurology, Children's Hospital of Nanjing Medical University, Nanjing, China; ^2^Clinical Metabolomics Center, China Pharmaceutical University, Nanjing, China; ^3^Department of Anesthesiology, Emory University School of Medicine, Atlanta, GA, United States

**Keywords:** intestinal surgery contributes, acute cerebellar ataxia, gut brain axis, gut flora, 16S rRNA

## Abstract

**Introduction:** Acute cerebellar ataxia (ACA) is the most common form of pediatric ataxia. Changes in gut flora can modulate the nervous system, influencing brain function via the gut-brain axis (GBA). This study aimed to illustrate the relationship between intestinal microbiota and ACA.

**Method:** A total of 30 and 12 children were randomly sampled from history of intestinal surgery (HOIS) and no intestinal surgery groups (NHOIS), respectively. In addition, 10 healthy children who sought physical examination in Children's Hospital of Nanjing Medical University were recruited as a control group. The stool samples were 16S rRNA detected.

**Results:** We observed that many ACA children had intestinal surgery history prior to the onset of ACA. The 16S rRNA sequencing indicated that HOIS and control groups were well-distinguished by principal component analysis. The discrepancy between HOIS and NHOIS groups were also displayed by principal component analysis score plot. However, no differences were found between NHOIS and control groups. The results of student's *t-*test were consistent with principal component analysis. A total of nine different genera were identified between HOIS and control groups. Five genera and a phylum showed significant differences between HOIS and NHOIS groups.

**Conclusion:** Altered genera and phyla associated with ACA were identified. Our findings provide new insight into treating and preventing ACA.

## Introduction

Acute cerebellar ataxia (ACA), is the most common form of pediatric ataxia, mostly affecting children 1–4 years old. ACA is non-genetic, and usually resulted from infections or vaccinations (1). In addition, neuroimmune diseases, poisoning, genetic metabolic diseases, cerebrovascular diseases, paraneoplastic syndromes, and other causes can trigger ACA. The symptoms of ataxia are obvious in trunk and lower limbs, including an inability to stand, unstable gait, falling easily, tremulous limbs, head, and body, unstable finger movement, inability for rotation action, and poor distance (2). While ACA is relatively benign and generally associated with a complete recovery (2, 3), several reports of severe or fatal outcomes have been described (3–8). These cases lasted for extended periods of time or became sequelae, impeding the intellectual development of children and increasing the risk of acute accidents (2).

Within the human intestine, trillions of microbes coexist symbiotically, influencing normal physiology and altering the host's susceptibility to disease (9). Increasing evidence in animals suggests that changes in gut flora or intestinal exposures can modulate the peripheral and central nervous systems, influencing brain function via a pathway called gut-brain axis (GBA) (10–14). The GBA involves neural, hormonal, and immunological signaling between the gut and the brain (15), and provides the intestinal microbiota and its metabolites with a potential route through which to access the brain (12).

Interestingly, we observed several ACA patients had undergone surgical bowel surgery prior to the onset of ACA. However, the relationship between intestinal microbiota and ACA has not been previously investigated. Hence, we hypothesized that intestinal surgery modulated the central nervous system via the GBA, leading to the onset of ACA. Consequently, this study was designed to investigate our theory and elucidate any alterations in intestinal microbiota. Our findings supported a gut-microbiome-brain connection in ACA and identified specific gut flora associated with ACA. As a result, this study provides a new insight into preventing and treating ACA.

## Methods

### Patients and Study Design

From January 2012 to April 2017, a total of 465 ACA children were enrolled from Children's Hospital of Nanjing Medical University and categorized into two groups: intestinal surgery group (*n* = 261) and without intestinal surgery group (*n* = 204). Relative clinical baseline characteristics were measured and recorded. Cerebellar ataxia includes genetic cerebellar ataxia and secondary cerebellar ataxia. The total cerebellar ataxia involved in this article is secondary ataxia, which is acute cerebellar ataxia in children.

Inclusion criteria included: (1) history of pre-infection, (2) acute onset (3) and the presence of ACA and associated symptoms, including manifested ataxia, often accompanied by limb tremor, nystagmus, decreased muscle tone, decreased sputum reflexes, and 4) the absence of other neurological diseases, systemic symptoms and other unclear neurological symptoms. We excluded patients with the following symptoms: (1) specific neurological infections, (2) drug poisoning, (3) congenital metabolic abnormalities, (4) posterior cranial fossa lesions, (5) hereditary dominant ataxia, and (6) infectious polyradiculitis or multiple sclerosis. Informed consent was obtained from all patients. This study was performed under the guidance of the Helsinki Declaration.

### Sample Collection and Preparation for 16S rRNA Sequencing

In order to investigate the role of gut microbiota in the ACA, 30 and 12 children were randomly sampled from history of intestinal surgery (HOIS) and no intestinal surgery groups (NHOIS), respectively. In addition, 10 healthy children who sought physical examination in Children's Hospital of Nanjing Medical University were recruited as a control group.

Before the commencement of any treatment, fecal samples were collected from the 52 children (30 HOIS, 12 NHOIS, and 10 controls subjects) described above. The stool samples of 52 subjects were collected. Their age and sex were matched. The middle part of the feces was collected, and divided into small portions. After collecting, the lid was closed immediately to make the samples in an anaerobic environment. The samples were placed under 0°C with ice box and transported back to the laboratory. The samples were store in a refrigerator at −80°C. A small portion was taken each time for extraction to avoid repeated freezing and thawing. The samples were placed in storage tubes and prepared for 16S rRNA detection.

### 16S rRNA Sequencing

DNA extraction and PCR amplification were performed using a DNA Stool Kit (Omega, CA, USA) according to the manufacturer's instructions. DNA yields were determined using a SpectraMax 190 (Molecular Devices, California, USA), and the integrity was evaluated via 1.0% agarose gel electrophoresis. DNA was then diluted to 1 ng·μL^−1^ in double distilled water.

Specific primers with barcode sequences were synthesized according to the designated sequencing regions. Low cycle number amplification was used to ensure consistent cycle numbers for sample amplification for accurate and consistent data analysis. A high-fidelity DNA polymerase (TaKaRa EX Taq) for PCR amplification. Thermal cycling consisted of denaturation at 94°C for 3 min, which was followed by 30 cycles of 94°C for 10 s, 55°C for 15 s and 72°C for 30 s, and finally held at 72°C for 7 min (Applied Biosystems 2720 Thermal Cycler). The amplified products were detected by 2% agarose gel electrophoresis and purified by magnetic beads using AxyPrep Mag PCR Clean-Up Kit (AXYGEN).

For 16S rRNA Illumina MiSeq sequencing, the sequencing libraries were constructed with an NEB Next Ultra DNA Library Prep Kit for Illumina (New England Biolabs Inc., Boston, MA, USA), and a Qubit 2.0 Fluorometer (Life Invitrogen, Inc., Carlsbad, CA, USA) was used to assess the libraries' qualities. The libraries were then sequenced on an Illumina MiSeq 2500 platform.

### Statistical Analysis

The continuous demographics characteristics were given as mean ± SD, while the categorical was given as percentages. Principal component analysis (PCA) was performed to estimate and visualize the differences between each two groups. Student's *t*-test was used to examine the differences between groups, whose *p* < 0.05 was considered statistically significant. All statistical analyses were performed using R software (version 3.3.3).

## Results

### Baseline Characteristics of Participants

A total of 465 ACA children were recruited from Children's Hospital of Nanjing Medical University and divided into two groups: intestinal surgery group (*n* = 261) and without intestinal surgery group (*n* = 204). The baseline characteristics of 465 children were presented in Table 1. In total, 102 boys in intestinal surgery group accounted for 39.08% and 91 boys in without intestinal surgery group accounted for 44.61%. The mean ages of the two groups were 32.61 ± 10.63 and 31.37 ± 12.96 months (*P* = 0.25), respectively. There were 85 (32.57%) and 37 (18.14%) children who relapsed in the HOIS and NHOIS groups, respectively (*P* = 6.65E-04).

**Table 1:** Baseline characteristics of 465 children with cerebellar ataxia.

	**All cerebellar ataxia (*****n*** **= 465)**		***P*-value**
	**HOIS (*n* = 261, 56.13%)**	**NHOIS (*n* = 204, 43.87%)**	
**Gender**			0.35
Boy	102	91	
Girl	159	113	
**Years old (months)**	32.61 ± 10.63	31.37 ± 12.96	0.25
**Recurrence (n, %)**	85 (32.57%)	37 (18.14%)	**6.65E-04**
**Infection (n, %)**	235 (90.03%)	186 (91.17%)	0.79
**MRI**			
Abnormal (%)	25 (9.57%)	19(9.31%)	0.94
Lower cerebellar tonsils	8	7	
Paraventricle white matter malacia	6	4	
Apical lower margin of the epencephal amygdala	5	3	
Cerebellar cistern cyst	4	3	
T2 signal shadow in the cerebellum	2	2	
**CSF examination**			
White blood cell (median)	10.08 (IQR:0–38)	8.94 (IQR:0–47)	0.52
Cl-(mmol/L)	121.91 ± 12.54	123.56 ± 13.28	0.17
Glucose (g/L)	3.52 ± 0.67	3.37 ± 0.53	**9.51E-03**
Protein (mmol/L)	0.16 ± 0.041	0.16 ± 0.032	0.99
Adenosine deaminase	1.66 ± 0.36	1.45 ± 0.49	**1.24E-07**
Lactic dehydrogenase	16.07 ± 3.54	15.37 ± 4.66	**1.71E-03**
**Electromyography**			
Abnormal (%)	28 (10.73%)	18 (8.97%)	0.59
Abnormal peroneal nerve	9	7	
Mild neurogenic impairment	15	8	
Multiple peripheral neurogenic lesions	4	3	
**Electroencephalogram**			
Abnormal (%)	10 (3.83%)	8 (3.92%)	0.85
Slow wave guide	2	2	
The bit slow background	4	3	
Frontotemporal apical wave	2	1	
The central midline sharp slow waves	1	1	
Borderline electroencephalogram	1	1	

*HOIS, history of intestinal surgery; NHOIS, no intestinal surgery groups; CSF, cerebrospinal fluid; IQR, interquartile range*.

*The statistically significant P-value are bold, which is smaller than 0.05*.

As for cerebro-spinal fluid (CSF) examination, significant differences existed in glucose (*P* = 9.51E-03), adenosine deaminase (*P* = 1.24E-07) and lactic dehydrogenase (*P* = 1.71E-03) between intestinal surgery and without intestinal surgery groups.

The detailed age distribution of children with ACA was described in Supplementary Table 1. Among these 465 children, 76 (16.38%) were under 12 months, 197 (42.46%) were 12–24 months, 106 (22.84%) were 24–36 months, 63 (13.58%) were 36–48 months, 15 (3.22%) were 48–60 months, and 8 (1.72%) were more than 60 months. The types of intestinal surgery of the 261 children with ACA were given in Supplementary Table 2. A total of 150 (57.47%) children underwent intussusception diorthosis, 42 (16.09%) indirect inguinal hernia hernioplasty, 15 (5.75%) appendicectomy, 17 (6.51%) intestinal torsion, 9 (3.45%) Meckel's diverticulum, 7 (2.68%) congenital duodenal atresia, and 21 (8.04%) other surgeries.

### 16S rRNA Sequencing

A total of 52 stool samples were allocated into HOIS, NHOIS, and control groups. The baseline characteristics of the 52 children were shown in Supplementary Table 3.

**Figure 1:**
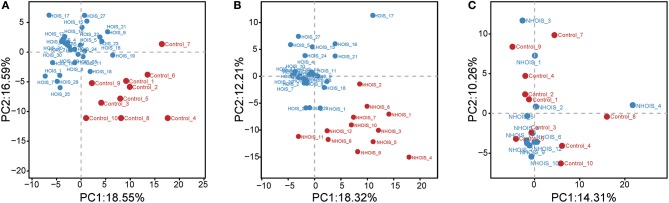
Principal component analysis score plots for comparations between HOIS, NHOIS and control groups. **(A)** The score plot HOIS vs. control. **(B)** The score plot between HOIS vs. NHOIS. **(C)** The score plot NHOIS vs. control.

Sequencing data were analyzed and visualized by PCA. Score plots were shown in Figure 1. Both HOIS and control (Figure 1A), and HOIS and NHOIS (Figure 1B) groups were well-distinguished. However, all samples randomly distributed without a clear connection between the NHOIS and control group (Figure 1C).

**Figure 2:**
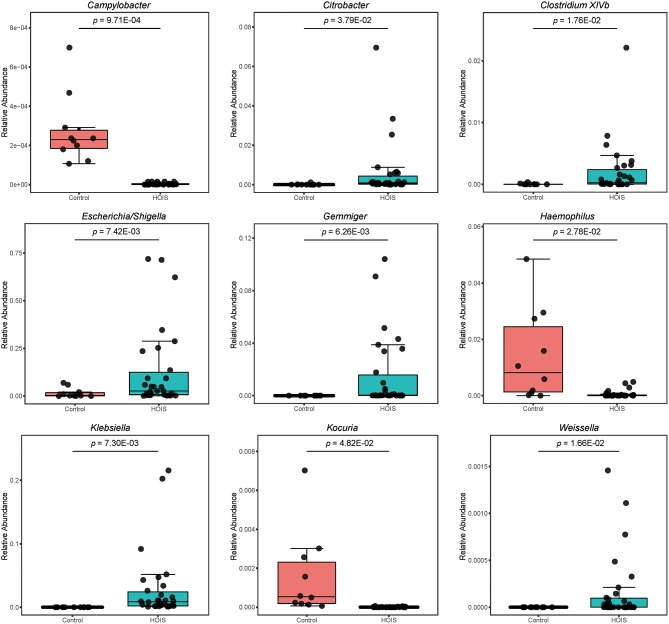
Boxplots for the genera and phyla significantly different between HOIS and control (*P* < 0.05).

Sequencing data were further compared between HOIS and control groups. In total, nine genera (*Campylobacter, Citrobacter, Clostridium XlVb, Escherichia/Shigella, Gemmiger, Haemophilus, Klebsiella, Kocuria*, and *Weissella*) were identified with significant differences, whose Student's *t p-*values were 9.71E-04, 3.79E-02, 1.76E-02, 7.42E-03, 6.26E-03, 2.78E-02, 7.30E-03, 4.82E-02, and 1.66E-02, respectively (Figure 2). Compared with the control group, the abundances of these distinct genera were elevated in HOIS group, except for *Campylobacter, Haemophilus*, and *Kocuria*.

**Figure 3:**
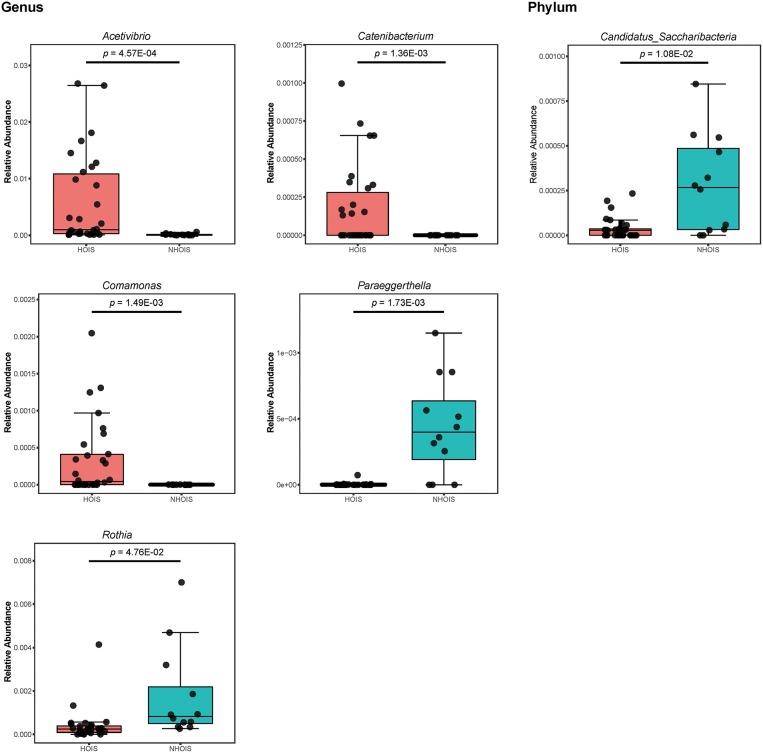
Boxplots for the genera and phyla significantly different between HOIS and NHOIS (*P* < 0.05).

The abundances in the significantly different genera and phyla between HOIS and NHOIS groups were shown in Figure 3, including five genera (*Acetivibrio, Catenibacterium, Comamonas, Paraeggerthella*, and *Rothia*) and phylum (*Candidatus Saccharibacteria*). Their Student's *t p*-values were 4.57E-04, 1.36E-03, 1.49E-03, 1.73E-03, 4.76E-02, and 1.08E-02, respectively. Compared with HOIS, the abundances of *Paraeggerthella, Rothia*, and *Candidatus s*. were greater in the NHOIS group, while *Acetivibrio, Catenibacterium*, and *Comamonas* showed lower abundances in NHOIS group.

No significantly distinct genus or phylum were found between NHOIS and control groups (data not shown). The results of *t-*test agreed with PCA. In addition to the genera and phyla described above, two genera and one phylum varied significantly between HOIS and control groups (data not shown), which have not been classified yet.

## Discussion

This study found that intestinal surgery may be an important risk factor for ACA. To confirm whether intestinal surgery is associated with the development of ACA via the GBA, this study sequenced fecal samples of 52 children. The gut flora discrepancies among the HOIS, NHOIS, and control groups were compared. Distinct florae were also identified, and their contributions were estimated.

A few studies have reported alterations of intestinal microbiota after intestinal surgery. In a randomized, double-blind trial involving 140 perioperative patients, decreased abundance of most gut flora induced by colorectal surgical stress were observed. Meanwhile, the abundance of some opportunistic pathogens and commensal microbiota increased (16). In our study, children in HOIS group underwent various surgeries, including intussusception, inguinal hernia repair, appendectomy and so on. Considering low similarity of these surgeries, we deduce surgery stress contributes to the change of intestinal flora. However, whether the alteration of gut flora is proportional to severity of ataxia remains unclear. The underlying mechanism how surgery contributing to gut flora needs further exploration.

As expected, our results showed significant difference between the HOIS and control groups, and NHOIS and HOIS groups. The distinct intestinal microbiota confirmed our hypothesis that intestinal surgery may affect gut flora in composition and abundance, leading to ACA. To avoid ACA induced by surgical stress, the abundance and diversity of gut flora for children are supposed to be measured. Recovery and maintainment of gut flora dynamic balance may contribute to preventing and treating ACA. Further preclinical and clinical studies are warranted.

Interestingly, no significant heterogeneity was observed between control and NHOIS groups. Compared to control, significantly distinct CSF protein, CSF adenosine deaminase, CSF lactic dehydrogenase, and significantly higher recurrence of ACA are observed in the intestinal surgery group. It indicated that the pathogenesis of ACA children with and without surgery are different. Current research suggests inflammation caused by a variety of acute infections as the most common cause of ACA (4, 17, 18). To our knowledge, no publication to date has indicated the contribution of gut flora on ACA. The role of GBA in neuropsychiatric disorders has been widely reported (19). During last decade, GBA was suggested to exert a profound influence on neural development, neuroinflammation, activation of stress response and neurotransmission (20). Patients subjected from neuropsychiatric disorders such autism spectrum disorders, schizophrenia, major depressive disorder and multiple sclerosis can have altered gut flora (21–24). It is reasonable to consider abnormal gut flora as a cause of ACA.

Microbiota can influence directly or indirectly via immune system activation, production of neurotransmitters, short-chain fatty acids and key dietary amino acids as tryptophan and its metabolites (12, 25). For instance, an altered gut flora can activate immune system (26), and start local and peripheral pro-inflammatory state, leading to a more permissive intestinal barrier. It makes bacteria mediators and pro-inflammatory easy to leave the gut reaching the brain and inducing neuroinflammation (27). Altered microbiota may result in ACA through above pathways.

The identification and study of microbial species relied then on their cultivation and phenotypic characterization for centuries. The birth of metagenomic sequencing facilitated an explosion of microbiome studies, enabling much of a microbial community to be identified in a single experiment. A few years later, other omic technologies arose (metatranscriptomics, metaproteomics, meta-metabolomics, etc.) to complement metagenomics, expanding the landscape of tools available for the high-throughput analysis of complex biomes (19, 28, 29). For our study, 16S sequence was not enough to uncover the underlying mechanism that gut microbial ecosystem participated in the cerebellar ataxia. The integration of multi-omics data into the “trans-omic” pipeline is able to generate unprecedentedly complete results. The analysis can get increasingly exhaustive catalogs of species, expressed genes, or metabolites.

## Limitations

Known limitations are presented as following: first, restricted samples were sequenced. A relatively small sample size may produce biased results. Second, because the sequencing data before ACA is absent, whether or not the baseline levels of intestinal microbiota were consistent was unknown. Additionally, GBA is a bidirectional communication system, enabling the brain to influence gastrointestinal functions, so the casual relationship remains to be further investigated. Third, due to restricted classified bacteria, unclassified bacteria perhaps play a key role in the development of ACA. Incremental classified bacteria may provide more complete insight into illustrating the role of gut flora in ACA.

## Conclusion

Intestinal surgery changed gut flora to contribute to ACA via the GBA, thus highlighting a novel pathogenesis of ACA. Altered genera and phyla involved in ACA were identified. Our findings provided a new insight into treating and preventing ACA.

## Data Availability

The raw data supporting the conclusions of this manuscript will be made available by the authors, without undue reservation, to any qualified researcher.

## Ethics Statement

The studies involving human participants were reviewed and approved by Nanjing Medical University. Written informed consent to participate in this study was provided by the participants' legal guardian/next of kin.

## Author Contributions

GZha, GZhe, and HG supervised this study. GZha, GZhe, GM, and SK conceived the idea of the manuscript. JY, YF, LW, YH, and LD drafted the first version of the manuscript. JY, C-FW, and XL conducted the experiment and statistical analysis. All authors revised manuscript, provided important intellectual content, and gave their final approval of the version submitted for publication.

### Conflict of Interest Statement

The authors declare that the research was conducted in the absence of any commercial or financial relationships that could be construed as a potential conflict of interest.

## Abbreviations

ACA: Acute Cerebellar Ataxia
CSF: Cerebro-Spinal Fluid
GBA: Gut-Brain Axis
HOIS: History of intestinal surgery
NHOIS: No intestinal surgery groups
PCA: Principal Component Analysis.
