# Supplementary material for: Intestinal Surgery Contributes to Acute Cerebellar Ataxia Through Gut Brain Axis
Source: Front Neurol. 2019 Sep 20;10:995. doi: 10.3389/fneur.2019.00995 (PMC6764330; doi:10.3389/fneur.2019.00995)
Supplement: Supplementary file 1 [file Table_1.DOCX]

Supplementary Table 1. The age distribution of children with cerebellar ataxia

| **Years old** | **Total (*n* = 465)** | **Ratio (%)** |
| --- | --- | --- |
| <12 months | 76 | 16.38 |
| 12-24 months | 197 | 42.46 |
| 24 -36 months | 106 | 22.84 |
| 36-48 months | 63 | 13.58 |
| 48-60 months | 15 | 3.22 |
| >60 months | 8 | 1.72 |

Supplementary Table 2. The types of Intestinal surgery for children with cerebellar ataxia

| **Surgery** | **Total (*n* = 261)** | **Ratio (%)** |
| --- | --- | --- |
| Intussusception diorthosis | 150 | 57.47 |
| Indirect inguinal hernia hernioplasty | 42 | 16.09 |
| Appendicectomy | 15 | 5.75 |
| Intestinal torsion | 17 | 6.51 |
| Meckel's diverticulum | 9 | 3.45 |
| Congenital duodenal atresia | 7 | 2.68 |
| Other surgery | 21 | 8.04 |

Supplementary Table 3. Baseline characteristics of 52 subjects treated with 16S rRNA sequencing.

|  | HOIS (*n* =30) | NHOIS (*n* =12) | Healthy control (*n* =10) |
| --- | --- | --- | --- |
| **Gender** |  |  |  |
| Boy | 14 | 5 | 4 |
| Girl | 18 | 7 | 6 |
| **Years old (months)** | 30.98±9.69 | 32.02±11.88 | 31.55±10.64 |
